# Quantifying time-dependent structural and mechanical properties of UV-aged LDPE power cables insulations

**DOI:** 10.55730/1300-0527.3407

**Published:** 2022-02-03

**Authors:** Abdallah HEDIR, Ferhat SLIMANI, Mustapha MOUDOUD, Omar LAMROUS, Tahar TOUAM, Madjid TEGUAR, Abderrahmane HADDAD, Ali DURMUS

**Affiliations:** 1 Laboratoire des Technologies Avancées en Génie Electrique (LATAGE), Université Mouloud Mammeri, Ouzou, Algérie; 2 Quantum Physics and Chemistry Laboratory (LPCQ), Mouloud Mammeri University of Tizi-Ouzou, Ouzou, Algeria; 3 Unité de Recherche en Optique et Photonique (CDTA-UROP) Sétif, Algérie; 4 Laboratory of Advanced Technologies in Electrical Engineering (LATAGE), Mouloud Mammeri University, Ouzou, Algeria; 5 Advanced High Voltage Engineering Research Centre, Cardiff University, The Parade, UK; 6 Department of Chemical Engineering, Engineering Faculty, İstanbul University-Cerrahpaşa, İstanbul, Turkey

**Keywords:** UV radiations, LDPE, degradation, crosslinking, scission, aging

## Abstract

This paper reports effects of ultraviolet (UV) light radiation on the physicochemical, electrical and mechanical properties of low-density polyethylene (LDPE) cable insulating materials. Changes in structural and morphological properties of UV-aged samples were characterized by various analytical methods such as attenuated total reflection Fourier transform infrared spectroscopy (ATR-FTIR), X-ray diffraction (XRD), scanning electron microscopy (SEM) and atomic force microscopy (AFM). Additionally, elongation at break, tensile strength, dielectric strength, and optical properties were also evaluated. Changes in some physical properties of LDPE after exposing to UV irradiation clearly highlighted that the polymer underwent the structural degradation. In addition, it was also found that such degradation yielded both crosslinking and chain scission as two competing processes during UV aging.

## 1. Introduction

Thermoplastics, elastomers, and thermoplastic compounds are often used for the insulation of electric cables because of their low cost, easy processing, outstanding dielectric and mechanical properties and their good chemical resistance [1]. Among these polymers, low-density polyethylene (LDPE) is one of the most commonly used polymers in cables insulation because it is a highly durable polyolefin under ordinary conditions due to its molecular features such as high molecular weight, hydrophobic, inert and nonpolar nature, and lack of functionality [2]. However, like the majority of polymers, LDPE undergoes irreversible physical and chemical changes which accelerate degradation when subjected to various environmental stresses such as humidity, heat, electric current, and radiation. Degradation affects directly the material properties causing a decrease in the service life of LDPE cables [3,4].

The chemical reactions of crosslinking, chain scission, oxidation, and formation of polar groups under ionizing radiation affect the insulating properties of LDPE [5]. In addition, its surface properties could be significantly damaged which would further deteriorate the LDPE properties [6]. Indeed, quantification of physicochemical properties of polyethylene insulating materials subjected to ionizing radiations has been attracted great technical interest by researchers from various areas such as polymer science, electrical engineering, chemistry and physics because this subject could be regarded as an interdisciplinary topic. Murray et al. [7] indicated that electron beam irradiation led to several modifications in the mechanical and structural properties of LDPE. Sabet et al. [8] showed that LDPE crystallinity decreased upon electron beam irradiation. Lanfranconi et al. [9] also studied isothermal crystallization of radiation-crosslinked LDPE and reported that the increase in the dose of radiation retarded the crystallization but, an increase in the oxygen content accelerated the crystallization of LDPE. They also illustrated that the crystallization activation energy increased with the radiation dose and decreased with the oxygen content. Sadighzadeh et al. [10] reported the effect of gamma radiation on elastic modulus and melting temperature of LDPE.

Overall, the UV is one of the most damaging radiations affecting the properties of the polymer materials. In fact, UV is a component of sunlight or some artificial sources such as solariums. The sun emits UV radiation across a broad spectrum from UV-C (wavelengths below 280 nm), and the UV-B band (280–315 nm) to UV-A band (315–400 nm) [11]. Note that UV-A represents approximately 95% of the UV radiation reaching the earth’s surface [12]. UV-exposure could provoke the initiation of oxidation reactions upon organic molecules under oxidative atmosphere by engendering a significant increase of the number of carbonyl groups (C=O) in the structure during photooxidation [13]. This photooxidative effect leads to higher incidence of morphological changes causing a deterioration of mechanical properties of polymers [14] and may conduct to the scission of covalent bonds on polymer chains randomly and substantial decrease in molecular weight [15]. Effects of UV radiation on the structural, physical and mechanical properties of polyolefins (e.g., LDPE, high density polyethylene, HDPE, linear low density polyethylene, LLDPE, and polypropylene, PP) have been studied [16–19]. Fairbrother et al. [20] studied the effects of temperature and light intensity on photodegradation of HDPE and reported that UV-exposure yielded a rapid embrittlement of HDPE was concurrent with increases in yield strength, stiffness, oxidation, and crystallinity. Rodriguez et al. [21] showed connections between the macroscopically observed behaviors and microstructural changes of UV-aged LDPE films. They also emphasized competition between multiscale phenomena: chain scission, crosslinking, chemicrystallization, oxidation-induced cracking and mechanical damage at the meso and coarse scales. Therias et al. recently reported the influences of UV-light intensity and temperature on the photooxidation kinetics of LLDPE [22]. In addition to the studies about structural changes of polyolefins, some researchers also focused on the visco-hyperelastic-viscoplastic behaviors and fatigue properties of photodegraded polyolefins [23,24]. Despite the large number of investigations have been reported in this area, the degradation mechanisms of polymers under UV radiation have not been well understood yet because of the complexity of involved phenomena and unique behaviors of macromolecular structures depending on their chemical and thermal properties as well as degradative conditions.

This study has intended to analyze the impact of UV radiation on the physical properties of LDPE power cable insulation. To achieve this goal, series of experiments were carried on LPDE samples. To simulate the effects of solar UV, eight low-pressure vapor fluorescent lamps of 36 Watts were used for aging LDPE samples for 480 h. The light wavelength was in the range of 315–400 nm. Several analytical methods were used to characterize physicochemical, dielectric and mechanical properties of samples. Various analytical instruments, attenuated total reflection Fourier transform infrared spectroscopy (ATR-FTIR), X-ray diffractometry (XRD), scanning electron microscopy (SEM) analysis, atomic force microscopy (AFM) were used to examine changes in the chemical constitution and the morphology of the aged LDPE material. Mechanical tests and breakdown characteristic measurements were also conducted to evaluate the material weakness and electrical rigidity of samples.

## 2. Experimental

Test specimens were prepared as square plates with a size of 130 × 130 mm and a thickness of 2 ± 0.2 mm with compression molding method by using a commercial grade of LDPE (Alcudia CP-104, Repsol Company) having a melt flow index (MFI) value of 2.4 g/10 min. (ASTM D1238) and density of 0.920 g/cm^3^.

The UV aging of the LDPE samples was performed in an accelerated UV aging chamber. The irradiation was accomplished using eight low-pressure vapor fluorescent lamps of 36 Watts. The irradiation wavelength is in the range of 350–400 nm. The distance between samples and lamps was 10 cm. The total aging time was 480 h. The physicochemical characterizations were evaluated by testing the specimens exposed to UV light for 240 and 480 h. However, the other characteristics were measured after each 48 h of exposure. The UV-exposure was performed at 55 ± 5 °C without controlling humidity.

In order to monitor the structural changes in LDPE due to UV-induced chemical issues, ATR-FTIR spectra were measured using a SHIMADZU spectrophotometer model IRAffinity-1S (Sanjo, Japan) with a resolution of 0.5cm^−1^. X-ray diffraction (XRD) analysis was also carried out to quantify possible microstructural changes in crystalline part of samples. For this purpose, Cu-Kα radiation in PANalytical X’Pert PRO (Malvern, United Kingdom) X-ray diffractometer was used.

The samples morphology was examined by scanning electron microscopy (SEM, JEOL JSM 6830) operating in clean vacuum. SEM analysis was performed under a voltage of 1 kV with × 500 magnification. In addition, atomic force microscopy (AFM) was used to determine surface roughness and topography of virgin and UV-aged LDPE samples. The experiment was performed in contact mode by a Nanosurf Flex-AFM system (Liestal, Switzerland) equipped with a 10 μm × 10 μm high-resolution scanner. AFM images were scanned with a resolution of 256 × 256 pixels over an area of 9 μm × 9 μm. Image processing and root mean square roughness (Rrms) calculations were achieved using the Gwyddion software. Finally, since color change is an indicator of polymer degradation, photographs of virgin and UV-aged samples were taken by using a high definition camera. Tensile tests were performed to compare the elongation at break and tensile strength, as well to evaluate the general relaxation behavior of material under mechanical load using a Schnek-Trebel testing machine according to the IEC 60811.1.1 (International Electrotechnical Commission) standard. After each 48 h of exposure time, samples in the form of dumbbell-shaped of 5 cm length were tested under ambient temperature (25 ± 2 °C). For each aging period, eight different specimens were tested. Finally, an AC breakdown test system which can supply a continuously adjustable power frequency AC voltage from 0 to 100 kV was implemented and used. The flat electrode with a diameter of 6 mm and the 60 × 60 mm LDPE square test tube are both immersed in transformer oil to prevent bypass. The breakdown tests are carried out at room temperature under a uniformly increasing voltage of 2 kV/s. For each aging period, six different samples were tested.

## 3. Results and discussion

**Figure 1:**
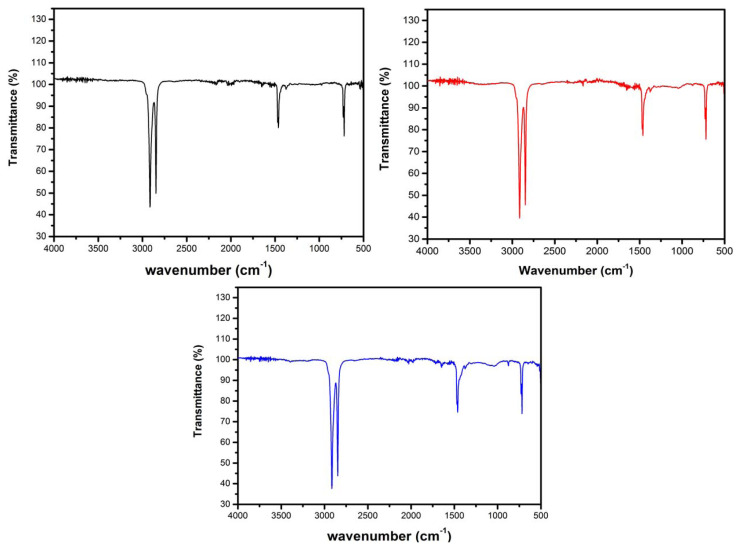
ATR-FTIR spectra of LDPE samples at different ultraviolet exposure times.

Figure 1 shows the FTIR transmittance spectra of unaged (virgin) LDPE (black), and aged LDPEs for 240 h (red) and 480 h (blue) recorded between the wavenumber of 4000 and 400 cm^−1^. Complete spectra illustrate well the characteristic bands of LDPE. The –CH_2_-stretching vibrations of main chain were observed at 2917 and 2846 cm^−1^ [25], while the bending and rocking vibrations of methylene (-CH-) units were appeared at 1471–1462 cm^−1^ and 731–719 cm^−1^ [26], respectively.

**Figure 2:**
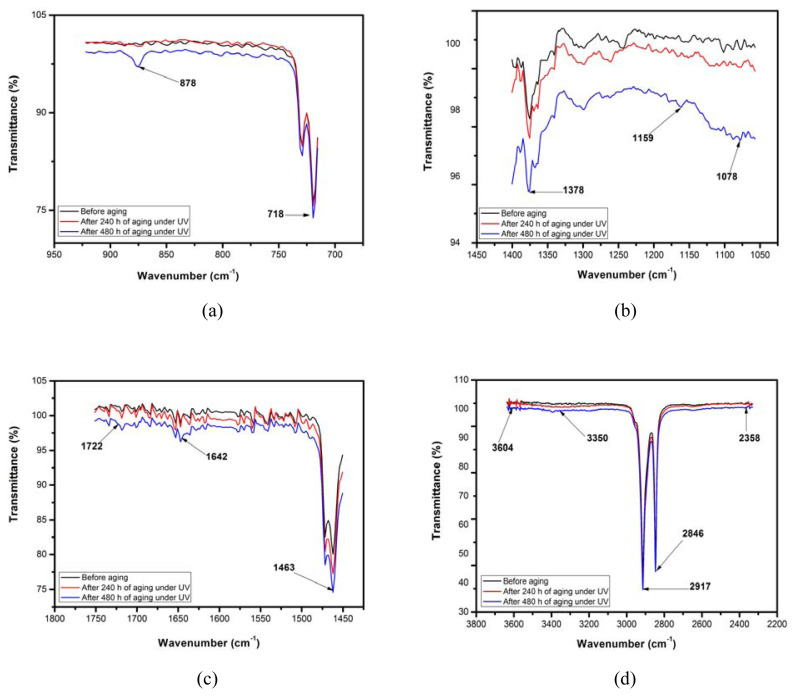
Enlarging view of the important characteristic peaks of FTIR spectra.

As shown in Figures 1 and 2, some small but significant changes in the intensities of characteristic bands of UV-irradiated LDPEs are observed compared to virgin LDPE. In fact, an increase in these intensities as a function of UV-radiation time is recorded for all of the chemical groups. Furthermore, on the evolution of IR spectra during the photoaging, formation of oxidation products characterized by the appearance of new peaks between 1200 and 800 cm^−1^ is remarkable. The absorption bands at 878 and 1159 cm^−1^ correspond to vinyl (-CH=CH_2_) [27] and alcohol (C-OH) [28] groups, respectively. The C-OH group could favor LDPE polarity and thus a dielectric strength decrease. The spectrum of UV-aged LDPE showed an absorption band at 1642 cm^−1^ due to carbon-C=O and carbonyl groups.

**Figure 3:**
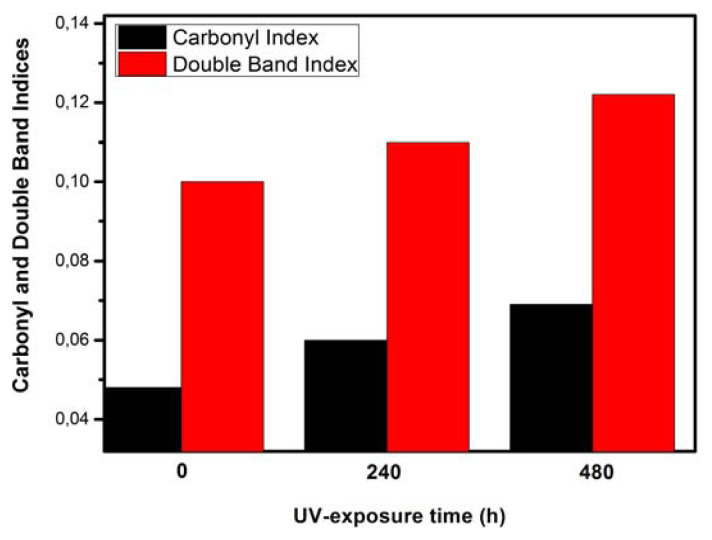
Carbonyl and double bond indices of LDPE samples as function of UV-exposure time.

In order to determine the oxidation level of LDPE after UV-irradiation, carbonyl index (CI) and double bond index (DBI) were calculated by using the ATR-FTIR spectra and the following formulas [29]:


$$CI=\frac{I_{1722}}{I_{1463}}\;\;\;\text{and }\;\;DBI=\frac{I_{1642}}{I_{1463}},$$

where *I_1722_* is the carbonyl (ketones) peak intensity at 1722 cm^−1^, *I_1463_* is the methylene peak intensity at 1463 cm^−1^, *I_1642_* is the vinyl group’s intensity at 1642 cm^−1^. Figure 3 represents the variations of both chemical groups’ indices as a function of aging-time.

**Figure 4:**
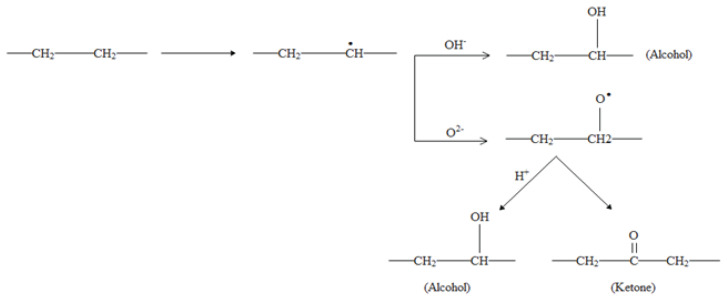
Oxidation scheme of LDPE.

From Figure 3, it can be noticed that the CI and DBI indices increased with the duration of UV aging. For instance, the values of CI and DBI indices were calculated to be 0.048 and 0.1, respectively for the virgin sample. Such values were 0.06 and 0.11 for the aging time of 240 h and 0.069 and 0.122 aging time of 480 h, respectively. The increase in the CI value is obviously related to the increase in the number of carbonyl groups which could be attributed to the photooxidation of LDPE according to the reaction mechanism of Norrish type II [30,31]. The increase in the CI during UV-exposure is consistent with the XRD results revealing that LDPE crystallinity increases with UV-exposure-time. This trend was previously noted by Salem et al. [32]. As can be seen in Figure 4, the photooxidation of LDPE can be achieved by the loss of a proton, leading to the formation of alcohol groups (C-OH). The obtained product is not stable. However, it can stabilize in two ways: (i) by the formation of an alcohol group via association with a proton, and (ii) by the formation of ketone (C=O) through the loss of the second proton linked to the carbon atom bonded to oxygen.

**Figure 5:**
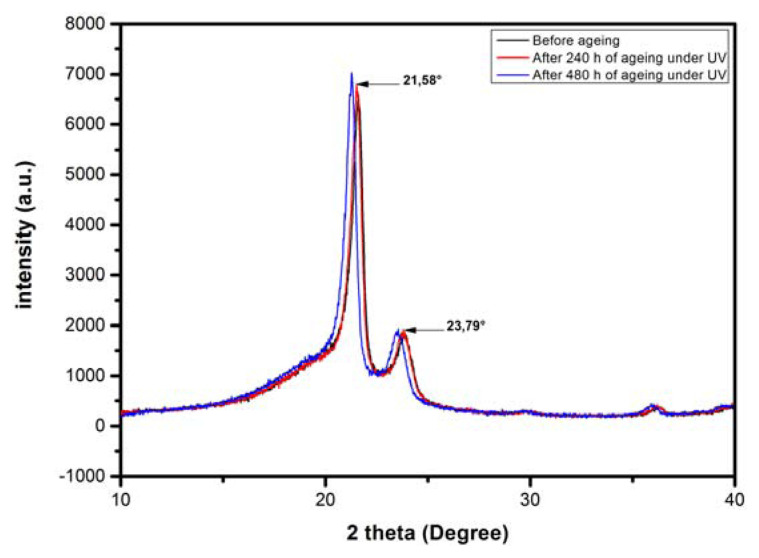
X-ray diffraction patterns of LDPE before and after ultraviolet exposure.

In the XRD patterns of samples presented in Figure 5, it is shown that both virgin and aged LDPE samples show roughly the same pattern. However, the X-ray spectra show a slight variation in the intensities of the peaks according to exposure time. Furthermore, these spectra show a very small deviation towards the left in the location of the peaks. As long as the deviation is very slight, it does not induce creation of significant new crystalline phases.

The crystallinity of LDPE was evaluated by using the Hinrichsen’s method and compared according to the UV-exposure time [33]. It consists of three Gaussian functions to fit the X-ray diffraction pattern, and the crystallinity index is calculated with the following formula:


$$\chi (\%)=\frac{Area2+Area3}{Area1+Area2+Area3}\times 100$$

where Area 1, Area 2and Area 3 denote respectively the areas of amorphous halo, principal crystalline peak at 2θ = 21.58°, and secondary crystalline peak at 2θ = 23.79° [33].

**Figure 6:**
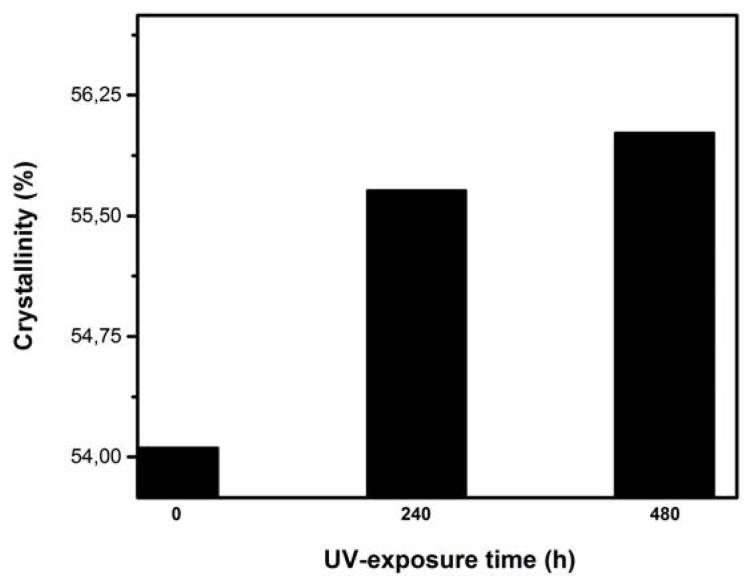
Crystallinity versus UV-exposure time.

Variation in the crystallinity index as a function of UV-exposure time is shown in Figure 6. The LDPE crystallinity increases gradually with the UV aging time. This increase can be related to the development of secondary crystallization [34], induced by the creation of shorter segments with greater mobility and created from chain scissions during the degradation process [35]. This change in crystallinity could also be attributed to the splitting of binding molecules crossing the amorphous regions followed by a rearrangement of the released segments for increasing the lamellar thicknesses of crystalline parts. This process favors the alignment of the incompletely crystallized chains at the manufacturing stage to increase crystallinity [29]. The X-ray results can be correlated with the mechanical behavior. Indeed, the slight decrease in tensile strength and elongation at break (see Figures 9 and 10) at the end of UV-exposure can be attributed to an increase in the LDPE crystalline phase.

**Figure 7:**
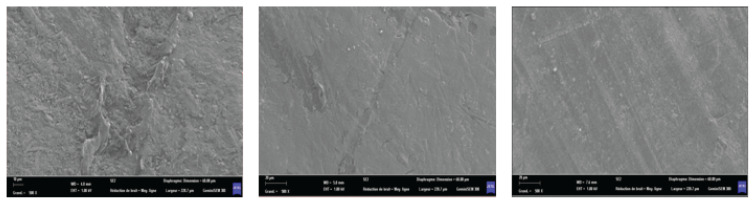
SEM images of virgin (a) and aged LDPE samples under 240 (b) and 480 h (c) of UV radiations. (× 500 magnification).

**Figure 8:**
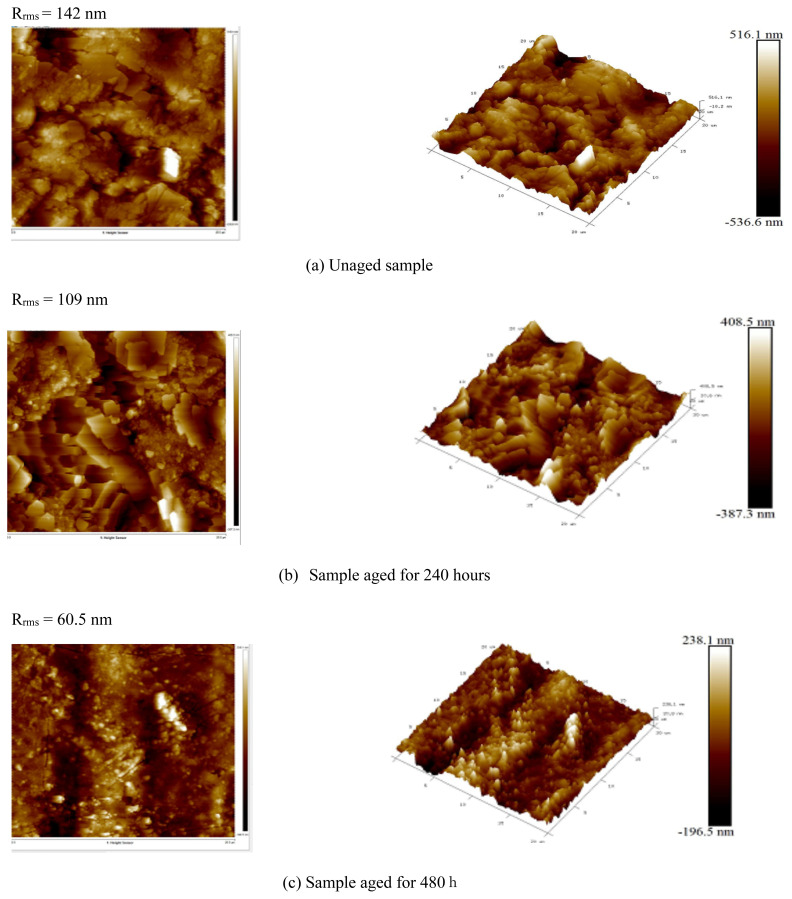
Measured 2D and 3D AFM images.

Figure 7 illustrates the SEM micrographs of virgin and UV-aged samples. With UV aging, the surface roughness of LDPE is reduced this decrease is mainly a result of the degradation (decrease in density) of the material. These results complement and confirm the AFM results (Figure 8).

Figure 8 shows the 9 μm x 9 μm two-dimensional (2D) and three-dimensional (3D) AFM topography images [36] of unaged and aged (480 h) samples. The effect of UV-exposure on the sample surface morphology is noticeably visible.

These images clearly illustrate that LDPE roughness profiles show a significant decrease. From Table, it can be seen that the root mean square roughness (Rrms) values measured for the unaged and aged samples are found to be 142 and 60.5 nm, respectively, which obviously indicate that the surface topography of the samples is strongly affected by aging. Therefore, UV rays impact leads to the crumbling of LDPE surface, which becomes smoother. This result may be attributed to the loss of impurities, moisture and residual solvent [4].

**Figure 9:**
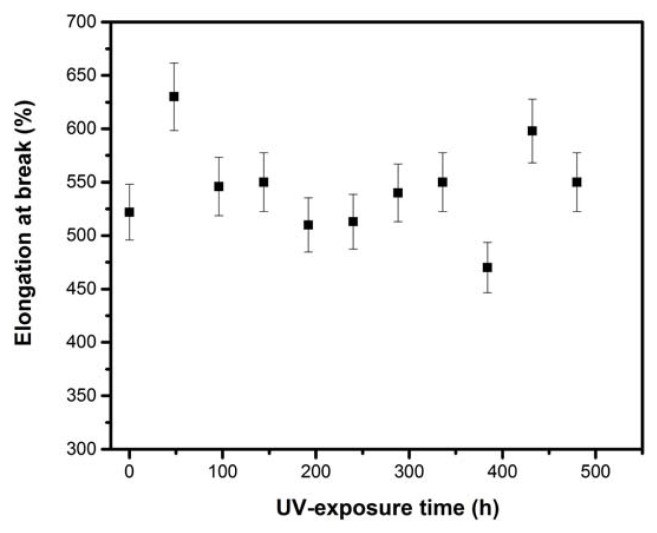
Variation of elongation at break versus UV aging time.

**Figure 10:**
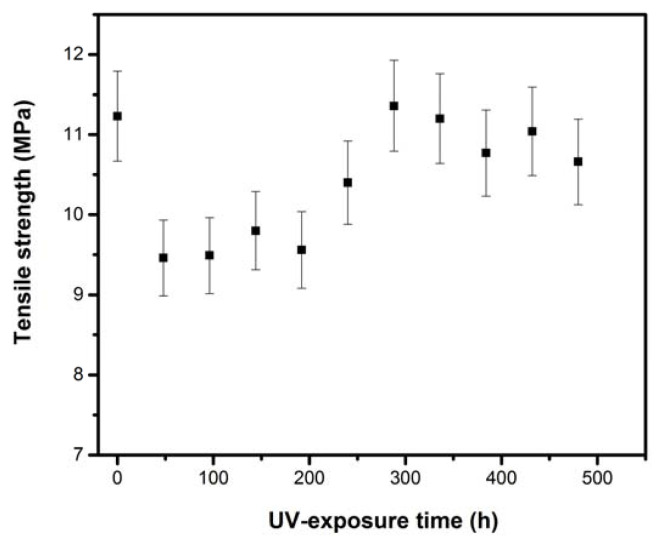
Tensile strength as function of UV aging time.

Figures 9 and 10 illustrate the evolutions of elongation at break and tensile strength versus UV-exposure time. The obtained results show that both elongation at break and tensile strength present nonmonotonic variation. Before UV-exposure, the elongation at break was 522%. This value increases to 630% after 48 h of UV-exposure. The changes in mechanical properties with the UV-exposure might be explained with the fact that increase physical and intermolecular interactions between PE chains due to increase in polarity (CI index) of the polymer occurring generally during the first UV-exposure phase, as reported by Skeikh et al. [37]. At the end of exposure, the elongation at break decreases to reach the value of 550%.

**Figure 11:**
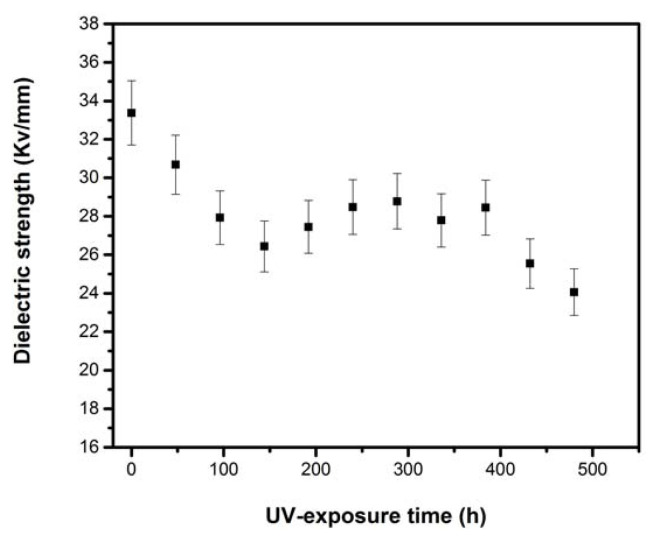
Dielectric strength versus UV aging time.

For tensile strength, the decrease is faster at the beginning of UV-exposure, where it dropped from 11.23 MPa to 9.46 MPa after only 48 h of exposure. After this time, the characteristic shows fluctuations up to 288 h and decreases gradually to reach the value of 10.66 MPa at the end of exposure. This behavior indicates that there is a competition between chains scission and crosslinking. The mechanical properties fluctuations over the whole studied period of irradiation, may be due to the cyclic effect of the photoinduced reactions of crosslinking/chain scission [32]. Indeed, the macromolecular chains will be subject to scission or to crosslinking reactions (chain recombinations), these two processes coexist generally concurrently or chronologically [38]. Perpetual chains orientation and increased orientation correlations explain the zigzag nature of elongation at break. The reorientations are slowed down by an increase in rigidity (see Figure 11), which decreases the dynamics of the chains at the local scale. The slight decrease in mechanical properties at the end of UV-exposure can be attributed to the induced chemical changes that simultaneously lead to a decrease in the amorphous zone size and in molecular weight. Indeed, these modifications cause weakness and embrittlement of the material.

Figure 11 presents the variation of dielectric strength versus aging time. As can be seen, LDPE shows a high initial dielectric strength of 33.4 kV/mm and then decreases rapidly with UV-exposure to reach 26.4 kV/mm after 144 h of exposure. Dielectric strength undergoes a slight increase in the interval of [144, 384] h, before falling to a minimum of about 24.1 kV/mm at the end of UV-exposure. From the beginning to the end of the aging test, a significant decrease in dielectric strength by 28% is recorded. This may be the result of the presence of different oxidative products such as the polar carbonyl and hydroxyl groups, originated by photooxidation during UV radiation aging [39].

**Figure 12:**
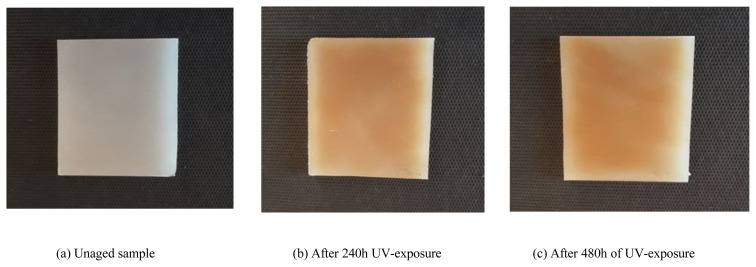
Changes in LDPE appearance with duration of UV-exposure.

Figure 12 shows the modifications in visual appearance of LDPE samples at different aging stages. It is observed that the color of the sample changes from grey, original color before aging, to light yellow after 240 h of UV-exposure and then to dark yellow after 480 h exposure. Also, it was noticed during the tests that the color concentration increases progressively with the exposure time. This behavior can be related to the phenomenon of photooxidation, which originates from the development of an oxidized layer on the surface of the insulating material [40]. Moreover, this change in color is induced by the formation of unsaturated groups such as vinylidene and vinyl groups after the chains breaking, also initiated by photooxidation. In the long term, these groups could induce a degradation of mechanical properties.

Our results agree well with those already made by Hiejima et al. [41]. These researchers showed that the color of LDPE samples changed from grey to yellow when increasing UV-exposure time. They have reported that the yellowness index can be used as an indicator for degradation of polymeric materials.

## 4. Conclusion

The main objective of this study was to follow and quantify the gradual degradation of LDPE samples submitted during 480 h under UV irradiation. Indeed, several characterization methods were used. FTIR techniques show the occurrence of an oxidation reaction because of the formation of carbonyl (C=O) and hydroxyl (OH) groups. The analysis of the FTIR spectra indicates the presence of C-OH groups. The most probable degradation mechanism giving such groups consists in chain scission. The influence of UV irradiation on the dielectric strength was also examined. A reduction of over 20% was recorded after only 144 h of UV-exposure. This behavior may be related to the presence of polar groups resulting from the photooxidation reaction. The LDPE mechanical properties are also affected as confirmed by XRD technique indicating the gradual increase of the material crystallinity during UV-exposure. Moreover, SEM and AFM show a decrease in surface roughness and the visual observations show that the LPDE color changes from grey to yellow during UV irradiation aging. Our investigation reveals that the crosslinking and chain scission are two competing mechanisms that coexist during aging degradation of the material.

## Figures and Tables

**Table:** Root mean square roughness (Rrms) evolution.

Sample	Root mean square roughness (R_rms_) (nm)
Before exposure	142.0
After 240 h of exposure	109.0
After 480 h of exposure	60.5
